# Functional regulation of the DNA damage-recognition factor DDB2 by ubiquitination and interaction with xeroderma pigmentosum group C protein

**DOI:** 10.1093/nar/gkv038

**Published:** 2015-01-27

**Authors:** Syota Matsumoto, Eric S. Fischer, Takeshi Yasuda, Naoshi Dohmae, Shigenori Iwai, Toshio Mori, Ryotaro Nishi, Ken-ichi Yoshino, Wataru Sakai, Fumio Hanaoka, Nicolas H. Thomä, Kaoru Sugasawa

**Affiliations:** 1 Biosignal Research Center, Organization of Advanced Science and Technology, Kobe University, Kobe 657-8501, Japan; 2 Graduate School of Science, Kobe University, Kobe 657-8501, Japan; 3 Friedrich Miescher Institute for Biomedical Research, CH-4058 Basel, Switzerland; 4 National Institute of Radiological Sciences, Chiba 263-8555, Japan; 5 Global Research Cluster, RIKEN, Wako 351-0198, Japan; 6 Graduate School of Engineering Science, Osaka University, Toyonaka 560-8531, Japan; 7 Advanced Medical Research Center, Nara Medical University, Kashihara 634-8521, Japan; 8 Faculty of Science, Gakushuin University, Tokyo 171-8588, Japan

## Abstract

In mammalian nucleotide excision repair, the DDB1–DDB2 complex recognizes UV-induced DNA photolesions and facilitates recruitment of the XPC complex. Upon binding to damaged DNA, the Cullin 4 ubiquitin ligase associated with DDB1–DDB2 is activated and ubiquitinates DDB2 and XPC. The structurally disordered N-terminal tail of DDB2 contains seven lysines identified as major sites for ubiquitination that target the protein for proteasomal degradation; however, the precise biological functions of these modifications remained unknown. By exogenous expression of mutant DDB2 proteins in normal human fibroblasts, here we show that the N-terminal tail of DDB2 is involved in regulation of cellular responses to UV. By striking contrast with behaviors of exogenous DDB2, the endogenous DDB2 protein was stabilized even after UV irradiation as a function of the XPC expression level. Furthermore, XPC competitively suppressed ubiquitination of DDB2 *in vitro*, and this effect was significantly promoted by centrin-2, which augments the DNA damage-recognition activity of XPC. Based on these findings, we propose that in cells exposed to UV, DDB2 is protected by XPC from ubiquitination and degradation in a stochastic manner; thus XPC allows DDB2 to initiate multiple rounds of repair events, thereby contributing to the persistence of cellular DNA repair capacity.

## INTRODUCTION

Genomic DNA continuously suffers damage from a plethora of sources, including the intrinsic instability of DNA, endogenously produced reactive oxygen species or other metabolites, and environmental agents such as radiation and chemical compounds. DNA damage interferes with DNA replication and transcription, and thereby induces mutations, chromosomal aberrations, cellular senescence, and apoptosis. Ultraviolet light (UV), one of the most common sources of DNA damage from the environment, induces characteristic dipyrimidinic DNA photolesions; e.g. cyclobutane pyrimidine dimers (CPDs) and pyrimidine–pyrimidone (6-4) photoproducts (6-4PPs). In mammals, these UV-induced photolesions are eliminated exclusively through a major DNA repair pathway, nucleotide excision repair (NER). Hereditary defects in NER are associated with several human autosomal recessive disorders, such as xeroderma pigmentosum (XP), which is clinically characterized by cutaneous hypersensitivity to sunlight and a remarkable predisposition to skin cancer (for recent reviews, see (1,2)).

A key step in DNA repair is the initial recognition of DNA damage. In mammalian NER, there are two sub-pathways, as follows: the global genomic NER (GG-NER) surveys the entire genome, whereas transcription-coupled NER (TC-NER) specifically removes RNA polymerase II-blocking lesions on template DNA strands during transcription. In particular, GG-NER decreases the frequency of replication forks that encounter DNA damage, thereby preventing mutagenesis and carcinogenesis. The XP-related protein XPC plays a crucial role (3–5). XPC forms a heterotrimeric complex with either of the two mammalian orthologues of *Saccharomyces cerevisiae* Rad23 (RAD23A and B) and centrin-2, a small calcium-binding EF-hand protein (6–8). In the cell-free NER reaction, the XPC complex functions as the initiator, and it has specific binding affinities not only for DNA containing a variety of helix-distorting base lesions, such as UV-induced 6-4PPs and bulky chemical adducts, but also for undamaged DNA containing mismatched bases (9,10). These biochemical studies, as well as a structural study of the *S. cerevisiae* XPC orthologue, Rad4 (11), have revealed that XPC/Rad4 indirectly senses structural abnormalities of DNA through interactions with the undamaged portion of the DNA duplex, in particular with the two ‘normal’ bases opposite the damaged site that oscillate due to impaired base pairing. Although this ‘indirect readout’ model for damage recognition plausibly explains the broad spectrum of substrate specificities associated with GG-NER, it also implies that XPC by itself is incapable of distinguishing whether damage that should be processed by NER is indeed present. This problem seems to be solved by a subsequent ‘damage verification’ step involving the basal transcription factor IIH (TFIIH) complex and the XPA protein, which prevents adverse incisions at sites devoid of damage. When TFIIH is recruited by DNA-bound XPC, two ATP-dependent helicase subunits, XPB and XPD, locally unwind the duplex DNA, allowing XPD (presumably together with XPA) to start translocation along a specific DNA strand in the 5’-3’ direction (12,13). The presence of damage is finally verified by blocking of XPD translocation, a process to which XPA may also contribute by recognizing a specific configuration of the complex containing kinked DNA (14,15). Upon verification of damage, other NER factors, such as replication protein A (RPA) and the two structure-specific endonucleases XPG and ERCC1-XPF, are recruited to accomplish dual incision and removal of damage (3).

Although XPC is responsible for the primary recognition of virtually all lesions within the huge repertoire of GG-NER substrates, UV-induced photolesions also recruit a specific additional factor for their detection and repair. The DDB1–DDB2 heterodimer, also designated as the UV-damaged DNA-binding protein complex (UV-DDB), specifically binds 6-4PPs with extremely high affinity and CPDs with moderate affinity (16,17), creating sites to which XPC is recruited (18–20). This mechanism is particularly relevant for the repair of CPDs, a type of damage associated with very limited DNA helix distortions that are prone to evade direct detection by XPC. By contrast, substantial removal of 6-4PPs occurs in the absence of UV-DDB, probably through direct recognition by XPC; however, UV-DDB has been proposed to stimulate GG-NER of 6-4PPs, especially when lesions are distributed sparsely throughout the genome, e.g. after irradiation with relatively low UV doses (19,21). The recently published crystal structure of UV-DDB revealed that DDB2, but not DDB1, is responsible for interaction with damaged DNA (22,23). In contrast to XPC, DDB2 has a C-terminal WD-repeat β-propeller domain that provides a hydrophobic pocket, which directly accommodates the two affected pyrimidine residues flipped out of the DNA duplex. In addition to its DNA-binding β-propeller domain, DDB2 has a structurally disordered N-terminal tail and an intervening helix–loop–helix motif that mediates interaction with DDB1. DDB1 contains three β-propeller domains, one of which interacts with the Cullin 4 (CUL4)–RBX1 ubiquitin ligase module to form the CUL4–RBX1–DDB1–DDB2 (CRL4^DDB2^) ubiquitin E3 ligase complex. DDB1 serves as a common adapter subunit for this ubiquitin ligase family (24–26), whereas DDB2 forms the substrate receptor and can be exchanged with other CRL4 substrate receptors, such as CSA (27) and CDT2 (28), to determine the substrate specificity of the ligase. The E3 ligase involving DDB2 is activated upon binding to UV-damaged chromatin, leading to ubiquitination of various nuclear proteins, including DDB2, XPC and histones H2A, H3 and H4 (29–31). This activation is accompanied by dissociation from the COP9 signalosome, a negative regulator of Cullin-containing E3 ligases and conjugation of NEDD8 to CUL4 (22,27).

The roles of the CRL4^DDB2^-mediated ubiquitination are somewhat controversial and remain to be understood. Several lines of evidence indicate that following UV irradiation, DDB2 undergoes poly-ubiquitination and degradation by the proteasome (32,33), for which lysine residues in the N-terminal tail are essential (22). In our hands, a majority of ubiquitinated XPC seems to escape degradation and revert to the unmodified form through deubiquitination (30), whereas other groups have argued that XPC undergoes substantial UV-induced degradation (34,35). Based on the observation that extensive poly-ubiquitination of DDB2 *in vitro* abolishes the remarkable damaged DNA-binding activity of UV-DDB, we proposed that CRL4^DDB2^-mediated ubiquitination is important for efficient handover of damage from UV-DDB to XPC and initiation of the subsequent NER reaction (30,36). Although a very recent report suggested that VCP/p97 segregase modulates poly-ubiquitin chains on DDB2 and XPC to ensure efficient NER (37), questions persist concerning the biological significance of DDB2 degradation and XPC ubiquitination. In this study, we further investigated the roles for the CRL4^DDB2^-mediated ubiquitination, focusing especially on the damaged DNA-binding activity and degradation of DDB2. Our results provide a novel insight into the role played by ubiquitination in regulating functional interactions between the two GG-NER damage-recognition factors.

## MATERIALS AND METHODS

### Cell lines and cell culture

Human fibroblast cell lines WI38 VA13 (normal) and XP4PASV (*XPC*-deficient) were cultured at 37°C in Dulbecco's modified Eagle's medium (Nissui) containing 10% fetal bovine serum. High Five cells were cultured at 27°C in Ex-Cell 405 medium (SAFC Biosciences).

### Establishment of stably transformed cell lines

The cDNA encoding HA-DDB2 was inserted into the bicistronic expression vector pIREShyg (Takara Bio). Various mutations in DDB2 were introduced into this construct using the QuikChange Mutagenesis Kit (Agilent Technologies). These constructs were linearized and introduced into WI38 VA13 cells by electroporation using a Gene Pulser II (Bio-Rad). Stable transformants were selected by culturing cells in the presence of 200 μg/ml hygromycin B (Life Technologies). XP4PASV cells expressing FLAG-XPC were established as described previously (30).

### Preparation of cell lysates

Cells in 60 mm dishes were washed twice with phosphate-buffered saline (PBS) and lysed *in situ* with 0.5 ml of ice-cold CSK buffer [10 mM Pipes–NaOH (pH 6.8), 3 mM MgCl_2_, 1 mM EGTA, 33.3% sucrose, 0.1% Triton X-100] containing 0.3 M NaCl, 10 mM *N*-ethylmaleimide (NEM; Wako Pure Chemicals), and protease inhibitor cocktail [0.25 mM phenylmethylsulfonyl fluoride (PMSF; Sigma–Aldrich), 1 μg/ml leupeptin, 2 μg/ml aprotinin, 1 μg/ml pepstatin and 50 μg/ml Pefabloc SC (AEBSF); all inhibitors, except for PMSF, were purchased from Roche Applied Science]. After incubation on ice for 1 h, the cell lysates were scraped into microfuge tubes and centrifuged for 10 min at 20 000 × *g* to obtain soluble extracts. The resultant pellets were resuspended with the aid of sonication in 0.25 ml of the same buffer.

### Immunoprecipitation

Soluble cell extracts were mixed with 30 μl of anti-HA (3F10) agarose beads (Roche Applied Science) and incubated overnight at 4°C. After the beads were extensively washed with CSK buffer containing 0.3 M NaCl, bound proteins were eluted with SDS sample-buffer [62.5 mM Tris–HCl (pH 6.8), 1% SDS, 10% glycerol, 0.02% bromophenol blue].

### Clonogenic cell survival assay

Two hundred cells were inoculated in a 100 mm culture dish and irradiated with various doses of UV-C under germicidal lamps (GL-15: Toshiba) with a 254-nm peak. At 21 days post-irradiation, the cells were washed twice with PBS, and then stained with 0.1% crystal violet in 10% ethanol for 30 min at room temperature. The stained dishes were washed with water, and colonies were counted.

### Purified protein factors

The XPC–RAD23B complex, centrin-2 and the DDB1–DDB2 complex were purified essentially as described previously (30,38). Recombinant baculovirus expressing His-tagged human UBA1 protein (E1 for ubiquitination) was generated using the Bac-to-Bac baculovirus expression system (Life Technologies). Twenty 150 mm dishes of High Five cells were infected with the baculovirus and incubated at 27°C for 3 days. The infected cells were then collected and resuspended in 80 ml of buffer containing 25 mM Tris–HCl (pH 8.0), 1 mM EDTA, 0.3 M NaCl, 1% Nonidet P-40, 10% glycerol, 1 mM dithiothreitol (DTT) and protease inhibitor cocktail. After incubation on ice for 30 min, the cell lysate was centrifuged at 20 000 × *g* for 20 min, and the resulting supernatant was dialyzed overnight against buffer containing 5 mM potassium phosphate (pH 7.0), 0.1 mM EDTA, 20 mM KCl, 10% glycerol, 1 mM DTT and 0.25 mM PMSF. The dialysate was centrifuged at 4°C for 30 min at 200 000 × *g* to yield clarified extract, which was loaded onto a HiTrap DEAE FF column (5 ml: GE Healthcare Biosciences) equilibrated with buffer A [5 mM potassium phosphate (pH 7.0), 10% glycerol, 0.01% Triton X-100, 1 mM 2-mercaptoethanol, 0.25 mM PMSF] containing 20 mM KCl. After washing with the same buffer, bound proteins were eluted with buffer A containing 0.5 M KCl. The eluate was then loaded onto a HiTrap Chelating HP column (1 ml: GE Healthcare Biosciences), which was pre-bound to nickel ions and equilibrated with buffer B [20 mM sodium phosphate (pH 7.8), 0.3 M NaCl, 10% glycerol, 0.01% Triton X-100, 1 mM 2-mercaptoethanol, 0.25 mM PMSF] containing 5 mM imidazole. After washing with the same buffer, the column was successively washed with buffer B containing 20, 100 and 250 mM imidazole. The 20 and 100 mM imidazole fractions containing His-UBA1 were combined, dialyzed against buffer C [25 mM Tris–HCl (pH 7.5), 1 mM EDTA, 10% glycerol, 0.01% Triton X-100, 1 mM DTT, 0.25 mM PMSF] containing 0.1 M NaCl, and further loaded onto a Mono Q HR5/5 column (GE Healthcare Biosciences) equilibrated with the same buffer. The column was developed with a linear NaCl gradient (12 ml) from 0.1 to 0.5 M in buffer C; His-UBA1 eluted around 0.2 M NaCl.

### Reconstitution of CRL4^DDB2^ E3 ligase

The His_6_-tagged complex containing human CUL4A (residues 38–759) and mouse RBX1 (residues 12–108) was expressed and purified by Ni-NTA, anion-exchange (POROS 50HQ; Life Technologies), and size-exclusion (HiLoad 16/60 Superdex 200; GE Healthcare Biosciences) column chromatography, essentially as described previously (22). The separately purified complex (30 μg) containing FLAG-DDB1 and DDB2 (wild type or mutant) was incubated on ice for 1 h with 90 μg of the CUL4A–RBX1 complex, and this mixture was then passed through a Superdex 200 PC 3.2/30 column (GE Healthcare Biosciences) equilibrated with buffer containing 40 mM Tris–HCl (pH 7.5), 1 mM EDTA, 0.1 M NaCl, 10% glycerol, 0.01% Triton X-100, 1 mM DTT and 0.25 mM PMSF. Eighty-microliter fractions were collected, and the fractions containing the four subunits were determined by SDS-PAGE followed by silver staining.

### Measurement of *in vivo* repair rates of UV-induced photolesions

Cells were cultured to 80% confluence in 100 mm dishes and maintained at 37°C for 2 h in medium containing 6 mM thymidine to prevent dilution of DNA lesions by replication. Cells were then irradiated with UV-C (at 10 J/m^2^ for 6-4PPs or at 2 J/m^2^ for CPDs) and cultured in the presence of 6 mM thymidine for the indicated times to allow DNA repair. Genomic DNA was purified with the QIAamp DNA Blood Mini Kit (Qiagen), and the levels of remaining photolesions were determined using an enzyme-linked immunosorbent assay with the lesion-specific antibodies (64M-2 or TDM-2; Cosmo Bio).

### *In vitro* ubiquitination assay

The standard reaction mixture (15 μl) contained 50 mM Tris–HCl (pH 7.5), 5 mM MgCl_2_, 0.2 mM CaCl_2_, 2 mM ATP, 1 mM DTT, 0.01% Triton X-100, bovine serum albumin (BSA; 1.5 μg), UV-irradiated plasmid DNA (2 kJ/m^2^; 100 ng), E1 (12.5 ng), UbcH5a (1 μg; Boston Biochem), ubiquitin (10 μg), purified CRL4^DDB2^ (50 ng) and XPC-RAD23B. The amount of ubiquitin was reduced where indicated. The reactions were incubated at 30°C for 30 min, stopped by addition of 1 μl of 0.5 M EDTA, and subjected to SDS-PAGE followed by immunoblot analyses using the appropriate antibodies.

### DNA-binding assay

Streptavidin-coated paramagnetic beads (Dynabeads M-280 Streptavidin; Life Technologies) were used to immobilize ligated arrays of a 30 mer double-stranded oligonucleotide containing a 6-4PP, as described previously (30). After *in vitro* ubiquitination reactions were carried out in the presence of these DNA beads, proteins bound or unbound to DNA were separated and subjected to immunoblot analyses.

### Antibodies, immunoblotting and immunostaining

Anti-XPC (13) and anti-RAD23B (39) antibodies were obtained as described previously. Anti-lamin B1 and CUL4 (Santa Cruz Biotechnology), anti-DDB1 (BD Biosciences), anti-DDB2 (R&D Systems) and anti-HA (3F10: Roche Applied Science) antibodies were purchased, respectively. For immunoblot analyses, proteins separated by SDS-PAGE were transferred onto Immobilon-P membranes (Merck Millipore) and detected by chemiluminescence using the appropriate secondary antibodies and substrates. Detection and quantitation were performed on an ImageQuant LAS-4010 biomolecular imager (GE Healthcare Biosciences). Blots were also exposed to X-ray films. Immunofluorescence staining was carried out basically as described previously (40).

### Mass spectrometry

Proteins were subjected to SDS-PAGE, followed by silver staining. The bands of interest were cut out and decolorized. After being alkylated with iodoacetamide, samples were digested with Sequencing Grade Modified Trypsin (200 ng; Promega) for 18 h at 37°C. The digested samples were analyzed on an LTQ Orbitrap Discovery mass spectrometer (Thermo Scientific), and the MASCOT software was used to determine ubiquitination sites.

### Other materials and methods

Silver staining was carried out with 2D-Silver Stain II Kit (Cosmo Bio) or, for mass spectrometry, the Silver Stain MS Kit (Wako Pure Chemicals). Cycloheximide (Sigma–Aldrich) and MG132 (Calbiochem) were purchased from the indicated suppliers.

## RESULTS

### Lysine residues in the DDB2 N-terminal tail are involved in cellular UV responses

**Figure 1.**
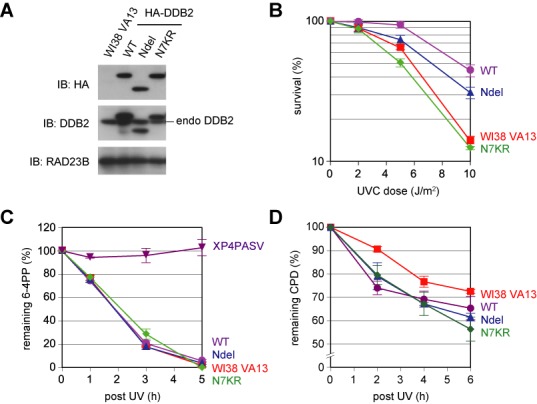
UV sensitivity and global genomic nucleotide excision repair (GG-NER) activity of transformed human fibroblast cell lines ectopically expressing wild-type or mutant DDB2 protein. (**A**) Immunoblot analyses of parental WI38 VA13 cells and transformed cell lines ectopically expressing various DDB2 proteins. RAD23B was used as a loading control. Note that reactivity of the anti-DDB2 antibody is slightly compromised by mutations in the N-terminal tail. (**B**) Clonogenic UV survival assay of the established cell lines. (**C** and **D**) Repair kinetics of UV-induced 6-4PPs (C) and CPDs (D). The *XPC*-deficient cell line (XP4PASV) was used as a negative control. For (B), (C) and (D), mean values and standard errors were calculated from three independent experiments.

The N-terminal tail of human DDB2 consists of approximately 100 amino acids, and seven lysine residues are present within the very N-terminal 40 amino acids. We previously reported that these lysines are targets for ubiquitination by CRL4^DDB2^ and are required for UV-induced degradation of DDB2 itself (22). In the preceding study, transformed cell lines were established from normal human fibroblasts (WI38 VA13), which stably expressed (in addition to endogenous DDB2) HA-tagged wild-type DDB2 (DDB2-WT) or mutant DDB2 with lysine-to-arginine substitutions at all seven lysines within the N-terminal tail (DDB2-N7KR). To further investigate the roles of the N-terminal tail, we established an additional cell line expressing HA-tagged DDB2 lacking the N-terminal 40 amino acids (DDB2-Ndel) (Figure 1A). As shown previously with DDB2-N7KR (22), DDB2-Ndel co-immunoprecipitated with DDB1 (Supplementary Figure S1) and CUL4A (data not shown) as efficiently as DDB2-WT, confirming that the N-terminal tail does not affect assembly of the CRL4^DDB2^ complex. Using these transformed cell lines, we examined the sensitivity to killing by UV. As shown in Figure 1B, ectopic expression of DDB2-WT conferred substantial UV resistance to the parental WI38 VA13 cells; this effect was slightly compromised by deletion of the N-terminal tail. Notably, ectopic expression of DDB2-N7KR did not confer any additional UV resistance on the parental WI38 VA13 cells (Figure 1B). As expected from the previous studies, the cell line expressing DDB2-WT removed UV-induced 6-4PPs from the genomic DNA at a comparable rate compared with the parental cells (Figure 1C), whereas repair of CPDs was slightly accelerated (Figure 1D). Similar repair kinetics was observed with the cell line expressing DDB2-Ndel or DDB2-N7KR, suggesting that the DDB2 N-terminal tail and, especially, the lysine residues within it are involved in regulation of cellular UV responses, independent of DNA repair activities.

### N-terminal lysines differentially affect UV-induced degradation and chromatin binding of DDB2

We previously showed that ectopically expressed DDB2-N7KR, but not DDB2-WT, is mostly resistant to UV-induced degradation mediated by the proteasome (22). On the other hand, poly-ubiquitinated DDB2 loses its damaged DNA-binding activity *in vitro*, suggesting that ubiquitination plays a role in dissociation of UV-DDB from sites of DNA damage, which we hypothesized may promote handover of damage to XPC (30). To investigate whether elimination of the major ubiquitination sites would affect the DNA-binding properties of DDB2 *in vivo*, the aforementioned cell lines were treated with UV and, at various time points, fractionated into soluble extracts and insoluble materials containing chromatin-bound proteins. Immunofluorescence analyses confirmed that the expressed HA-DDB2 proteins localized predominantly within the nucleus regardless of the presence or absence of mutation (Supplementary Figure S2).

**Figure 2.**
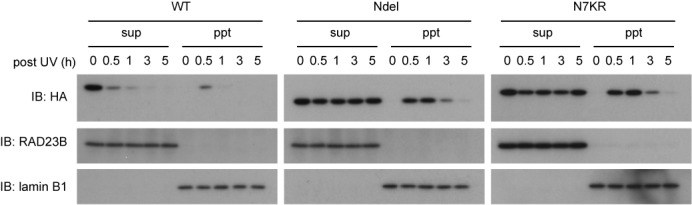
Mutant DDB2 proteins lacking the N-terminal lysines bind to chromatin, but resist UV-induced degradation. Transformed cell lines expressing indicated HA-DDB2 (WT, Ndel or N7KR) were pre-treated for 2 h with 1 mM cycloheximide (CHX), and then irradiated with UV-C at 10 J/m^2^. Following further incubation for various times in the presence of CHX, the cells were fractionated into soluble extracts (sup) and insoluble materials (ppt), which were then subjected to immunoblot analyses. RAD23B and lamin B1 were used to validate the fractionation, and also as loading controls.

When the ectopically expressed DDB2 was detected by immunoblotting with anti-HA antibody, all DDB2 species (WT, Ndel and N7KR) transiently associated with chromatin, peaking around 0.5–1 h after irradiation (Supplementary Figure S3). In response to UV, a slight decrease in protein levels was discerned for DDB2-WT, but not DDB2-Ndel or -N7KR. This difference was dramatically larger when similar experiments were carried out in the presence of cycloheximide to inhibit *de novo* protein synthesis (Figure 2). Although transient chromatin association of DDB2-WT was still observed, the total protein level was reduced so rapidly that it mostly disappeared by 3 h post-irradiation. By striking contrast, total levels of the two mutant DDB2 proteins appeared to persist even after UV irradiation, in line with our previous findings regarding DDB2-N7KR (22). Notably, as these mutant DDB2 proteins disappeared from the chromatin fractions, they reverted to the soluble fractions (most pronounced at 5 h post-UV). Taken together with the results in Figure 1C and D, these findings demonstrate that the N-terminal lysine residues of DDB2 appear to be dispensable for dissociation of UV-DDB from UV-damaged chromatin and normal NER processing.

### Identification of DDB2 ubiquitination sites outside the N-terminal tail

Our previous studies with cell-free ubiquitination assays indicated that, apart from the major sites identified within the N-terminal tail, DDB2 also contains a few additional sites that can be targeted by auto-ubiquitination *in vitro* (22). It is possible that poly-ubiquitination of DDB2 has different functions depending on the sites that are modified; poly-ubiquitination within the N-terminal tail may primarily be responsible for UV-induced degradation of DDB2, whereas poly-ubiquitination at other sites may regulate its interaction with damaged DNA. To test this possibility, we next tried to identify the ubiquitination sites in DDB2 outside the N-terminal tail.

For this purpose, we prepared recombinant CRL4^DDB2^ complexes, which contained either DDB2-WT or DDB2-Ndel in addition to FLAG-DDB1, CUL4A and RBX1 (Supplementary Figure S4A). These complexes were immobilized on anti-FLAG antibody beads and used for *in vitro* auto-ubiquitination reactions containing “K-less” mutant ubiquitin, in which all lysine residues were changed to arginines to prevent poly-ubiquitin chain formation. After the beads were washed extensively, bound proteins were eluted and subjected to SDS-PAGE, followed by silver staining. As shown in Supplementary Figure S4B, multiple bands corresponding to mono-ubiquitinated DDB2-Ndel were discernable, whereas modified DDB2-WT bands were difficult to identify due to the presence of a larger number of ubiquitination sites, resulting in overlapping bands on the gel. When mono-ubiquitinated DDB2-Ndel was digested with trypsin and analyzed by mass spectrometry, at least six lysine residues (K146, K151, K187, K233, K278, K362) were found to undergo the modification. Based on the diGly proteomics, it was reported that among these sites, at least K151 and K187 indeed undergo ubiquitination *in vivo* (41).

**Figure 3.**
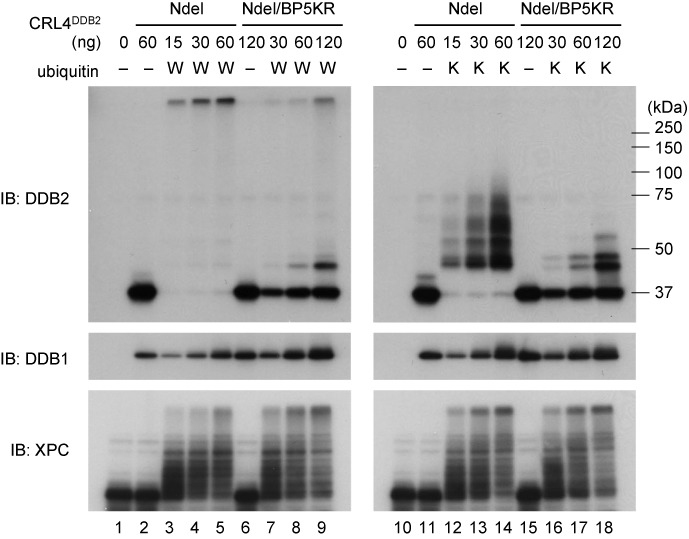
DDB2-Ndel/BP5KR protein is mostly resistant to self-ubiquitination *in vitro*. The indicated amounts of the CRL4 E3 ligase complex containing either DDB2-Ndel or DDB2-Ndel/BP5KR were tested in *in vitro* ubiquitination assays, with or without wild-type ubiquitin (W) or K-less mutant ubiquitin (K). The reactions were subjected to immunoblot analyses with the indicated antibodies.

The X-ray crystal structures of the CRL4^DDB2^ complex revealed that the catalytic center for ubiquitination, formed on the C-terminal tip of the rod-shaped CUL4, could freely move around within a certain range of space, the so-called “ubiquitination zone” (22). The newly determined ubiquitination sites reside within the β-propeller domain of DDB2 and, except for K362, five of them are expected to be inside the ubiquitination zone. Because it was unlikely that CUL4 could target K362 of DDB2 within the same CRL4^DDB2^ complex, we decided to introduce lysine-to-arginine substitutions at the remaining five sites in DDB2-Ndel (designated as DDB2-Ndel/BP5KR). The recombinant E3 ligase containing DDB2-Ndel/BP5KR was prepared and used for *in vitro* ubiquitination reactions. In line with our previous report (22), when normal ubiquitin was included in the reactions, DDB2-Ndel underwent extensive poly-ubiquitination and barely entered the gel (Figure 3, lanes 3–5). Under similar conditions, a majority of DDB2-Ndel/BP5KR remained unmodified, indicating that most of the ubiquitination sites in this mutant protein were deleted, as expected (lanes 7–9). However, a very small fraction of DDB2-Ndel/BP5KR still seemed to undergo mono- or poly-ubiquitination and parallel experiments with K-less ubiquitin revealed the presence of a few minor modification sites (lanes 16–18).

**Figure 4.**
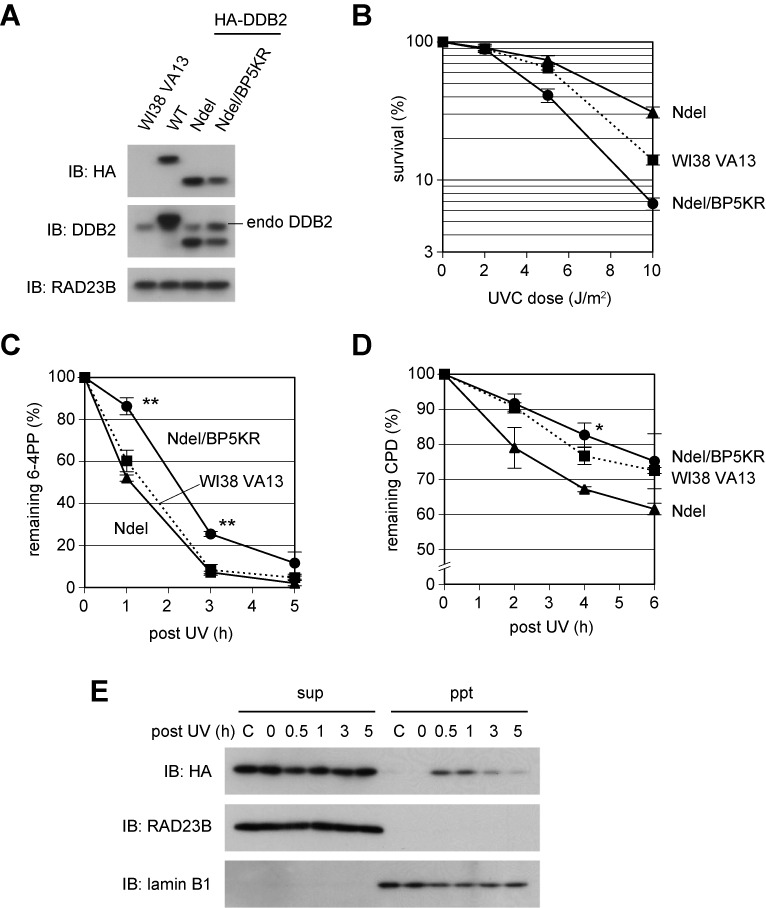
Non-ubiquitinatable DDB2 interferes with cellular GG-NER function. (**A**) Immunoblot analyses of a transformed cell line stably expressing HA-tagged DDB2-Ndel/BP5KR protein. The protein was expressed at a slightly lower level than DDB2-WT and DDB2-Ndel, while the level of endogenous DDB2 was elevated. RAD23B was used as a loading control. (**B**) Exogenous expression of DDB2-Ndel/BP5KR made cells more sensitive to UVC. (**C** and **D**) Repair kinetics of UV-induced 6-4PPs (C) and CPDs (D). The cells expressing DDB2-Ndel/BP5KR showed significantly slower repair of 6-4PPs (**P < 0.01) and CPDs (*P < 0.05) than in the DDB2-Ndel expressing cells. For (B), (C) and (D), mean values and standard errors were calculated from three independent experiments. (**E**) Behaviors of the DDB2-Ndel/BP5KR protein in response to UV irradiation. Cells were treated and analyzed as done in Figure 2. As a control, cells without cycloheximide (CHX) and UV treatment were analyzed in parallel (indicated by ‘C’ above the lanes).

To examine *in vivo* functions and behaviors of this ubiquitination-resistant DDB2, we established a transformed cell line from WI38 VA13, which stably expressed HA-tagged DDB2-Ndel/BP5KR (Figure 4A). As shown in Figure 4B, this cell line was slightly more sensitive to UV than the parental cells, although the ectopic expression of DDB2-Ndel conferred significant UV resistance. Furthermore, this cell line exhibited significantly slower repair of 6-4PPs following UV irradiation (Figure 4C), whereas the enhanced repair of CPDs with the DDB2-Ndel expression was also canceled (Figure 4D). Next the cells were exposed to UV and fractionated into soluble extracts and insoluble materials at various time points, as in Figure 2. DDB2-Ndel/BP5KR lacking the N-terminal tail was resistant to UV-induced degradation, as expected (Figure 4E and Supplementary Figure S5). Although it seemed to bind chromatin normally upon UV irradiation, DDB2-Ndel/BP5KR tended to reside longer on chromatin; however, the level of the chromatin-bound protein eventually decreased, suggesting that this mutant DDB2 protein was nonetheless able to dissociate from sites with DNA damage.

### Poly-ubiquitination abrogates damaged DNA binding by DDB2, regardless of which sites are modified

**Figure 5.**
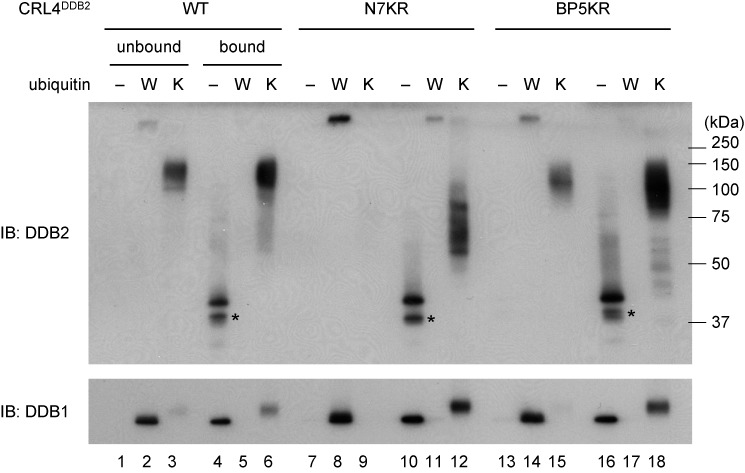
Poly-ubiquitination, but not mono-ubiquitination, abrogates damaged DNA-binding activity of DDB2 regardless of modification sites. The CRL4 E3 ligase complex containing the indicated DDB2 (WT, N7KR or BP5KR) was tested in *in vitro* ubiquitination assays in the presence of paramagnetic beads bearing DNA containing 6-4PPs. Reactions were performed in the absence of ubiquitin (−), or in the presence of wild-type ubiquitin (W) or K-less ubiquitin (K). Proteins bound or unbound to the DNA beads were separated and subjected to immunoblot analyses. Asterisks indicate degradation products of DDB2 generated during the incubations. DDB1 exhibits a slight band shift only in the reactions containing K-less ubiquitin (lanes 6, 12 and 18), suggesting that DDB1 is not targeted by CUL4 as long as conjugation sites are available on more efficient substrates, such as DDB2 and ubiquitin.

To determine the site at which DDB2 ubiquitination modulates damaged DNA-binding activity, two DDB2 mutant proteins were used for *in vitro* ubiquitination and DNA-binding assays; DDB2-BP5KR had the intact N-terminal tail but lacked the five ubiquitination sites in the β-propeller domain, whereas DDB2-N7KR lacked the seven lysines in the N-terminal tail, so ubiquitination of this protein could occur only in the β-propeller domain. CRL4^DDB2^ complexes containing one of these mutant DDB2 proteins were prepared and used for cell-free ubiquitination reactions in the presence of paramagnetic beads bearing immobilized DNA containing UV-induced 6-4PPs. After the reactions, DNA-bound and -unbound proteins were separated and detected by immunoblotting (Figure 5). As shown in our previous study (30), almost all of the unmodified DDB2-WT bound to the damaged DNA beads (compare lanes 1 and 4), whereas poly-ubiquitinated DDB2-WT was recovered mostly in the unbound fraction (lanes 2 and 5). When K-less ubiquitin was used instead of normal ubiquitin, DDB2-WT predominantly remained bound to damaged DNA (lanes 3 and 6), corroborating the idea that mono-ubiquitination cannot compromise the damage-recognition activity of DDB2, even when it occurs at multiple sites. Because essentially the same results were obtained with DDB2-N7KR (lanes 7–12) and DDB2-BP5KR (lanes 13–18), we conclude that poly-ubiquitination abolishes damaged DNA-binding activity of DDB2, regardless of which sites are modified. In a similar experiment, DDB2-Ndel behaved indistinguishably from DDB2-N7KR, whereas DDB2-Ndel/BP5KR poorly underwent poly-ubiquitination and thus tended to be retained by damaged DNA (Supplementary Figure S6).

### XPC negatively regulates UV-induced degradation of DDB2

**Figure 6.**
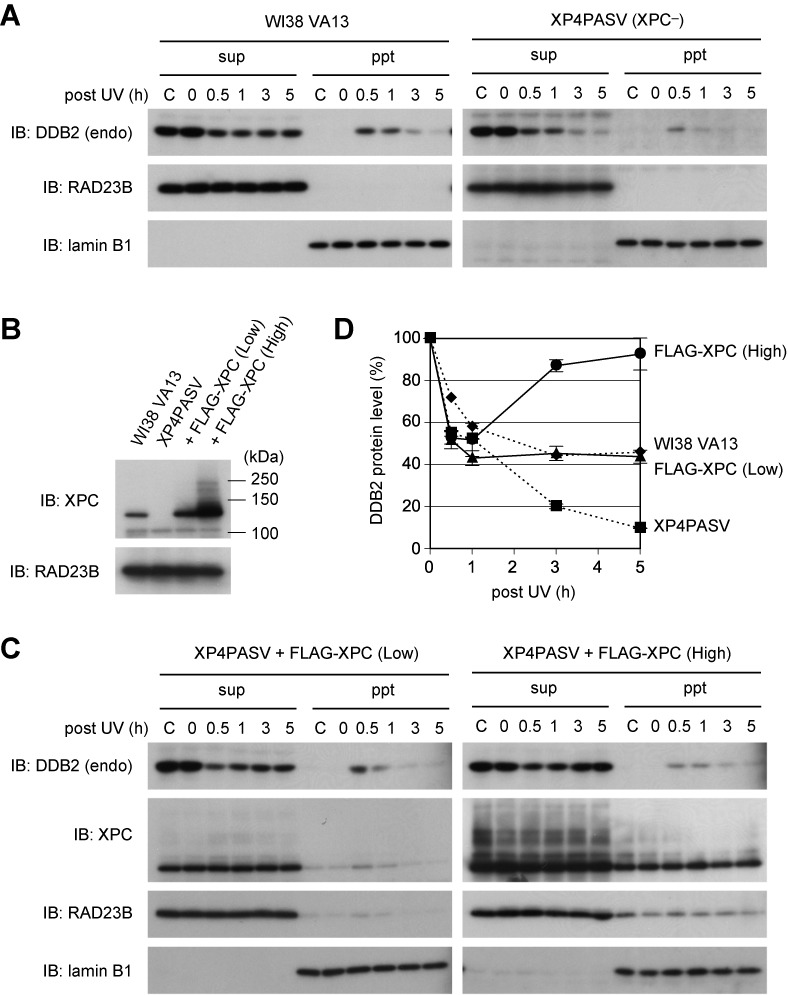
XPC suppresses UV-induced degradation of endogenously expressed DDB2 protein. (**A**) Behaviors of endogenous DDB2 in WI38 VA13 (normal) and XP4PASV (*XPC*-deficient) cells after UV-C irradiation at 10 J/m^2^. Cells were treated with cycloheximide (CHX) and fractionated as in Figure 2. (**B**) Immunoblot analyses of the transformed XP4PASV cell lines that stably expressed FLAG-XPC at different levels. RAD23B was used as a loading control. (**C**) Exogenous XPC expression suppressed UV-induced destabilization of endogenous DDB2 in XP4PASV cells. Treatment with CHX, UV irradiation and fractionation of cells were performed as in Figure 2. (**D**) Relative amounts of the endogenous DDB2 protein (as the sum of soluble extracts [sup] and insoluble materials [ppt] fractions) were determined from band intensities in (A) and (C), and plotted as a function of time after UV irradiation (DDB2 levels in the lanes labeled ‘0’ are defined as 100%). Mean values and standard errors were calculated from two independent experiments.

The results described above concerning ubiquitination-resistant DDB2 suggest that poly-ubiquitination of DDB2 is not essential for dissociation from UV-damaged chromatin, although it may promote dissociation. This observation prompted us to reexamine behaviors of endogenously expressed DDB2 in response to UV irradiation. When WI38 VA13 cells were treated with UV and fractionated as described above, we noticed that degradation of endogenous DDB2 was not as pronounced as that of ectopically expressed DDB2-WT, despite the presence of the intact N-terminal tail (Figure 6A, left panel). Even in the absence of *de novo* protein synthesis, a portion of chromatin-bound DDB2 reverted to the soluble extract at later time points. By contrast, in the *XPC*-deficient human fibroblast cell line (XP4PASV), endogenous DDB2 was extremely unstable after exposure to UV (Figure 6A, right panel). This UV-induced destabilization of endogenous DDB2 was similarly observed with XP4PASV cells that were not treated with cycloheximide (Supplementary Figure S7). To determine whether the observed difference in DDB2 stability could be attributed to the presence or absence of XPC, we took advantage of XP4PASV-derived transformed cell lines that stably expressed FLAG-XPC either at a nearly physiological level or at a much higher level (Figure 6B). In these cell lines, the UV-induced destabilization of endogenous DDB2 was dramatically alleviated, and this effect appeared to depend on expression levels of XPC (Figure 6C; see also quantitative data in Figure 6D). Furthermore, we have previously reported that the W690S mutation of XPC, identified from a patient with XP group C, completely abolishes its DNA binding activity, although this mutant XPC protein *in vivo* is still recruited to the sites with UV-induced DNA damage in a DDB2-dependent manner (42). When this mutant version of FLAG-XPC was stably expressed in XP4PASV cells, endogenous DDB2 showed similar instability upon UV treatment as observed in the parental cells (Supplementary Figure S8), indicating that the DNA binding activity of XPC is crucial to counteract the UV-induced destabilization of DDB2.

**Figure 7.**
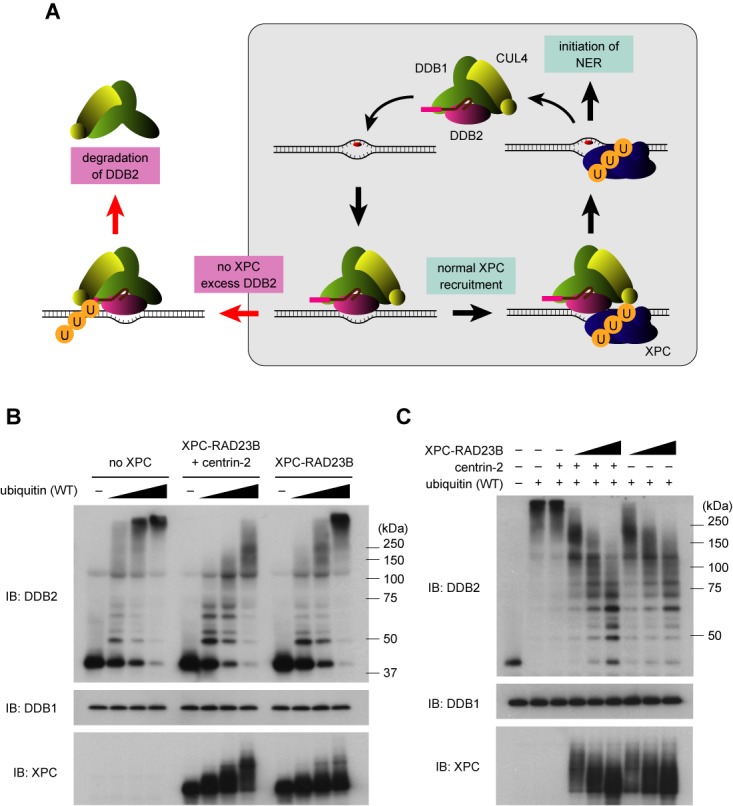
XPC competitively suppresses ubiquitination of DDB2. (**A**) A model for substrate selection by the CRL4^DDB2^ E3 ligase. As long as XPC is recruited correctly, CRL4 preferentially targets XPC so that DDB2 can escape degradation and recycle to initiate multiple rounds of NER; thus the stability of DDB2 may be regulated in a stochastic manner, depending on the balance between XPC and DDB2. (**B**) *In vitro* ubiquitination reactions were performed with the CRL4^DDB2^ complex (63 ng) in the presence of varied amounts (31, 63, 125 ng) of ubiquitin (wild type). The XPC–RAD23B complex (164 ng), with or without centrin-2 (164 ng), was included where indicated. After incubation, the reaction mixtures were subjected to immunoblot analyses with the indicated antibodies. (**C**) A similar experiment as (B) with a fixed amount of ubiquitin (63 ng) and varied amounts of XPC–RAD23B (82, 164, 328 ng). Centrin-2 (330 ng) was also added where indicated.

The results described above strongly suggest that DDB2 *in vivo* does not always undergo degradation even upon binding to UV-induced photolesions. As long as XPC is properly recruited, CRL4^DDB2^ bound to DNA damage may preferentially target XPC, allowing the N-terminal tail of DDB2 to escape poly-ubiquitination, which, in turn, enables DDB2 to be involved in multiple rounds of damage recognition (Figure 7A). To test this possibility, we next examined how XPC affects ubiquitination of DDB2 *in vitro*. When ubiquitin at sub-optimal concentrations was included in the reactions, the presence of XPC significantly suppressed ubiquitination of DDB2 (Figure 7B). Notably, this inhibitory effect of XPC was much more pronounced in the presence of centrin-2, which interacts with XPC and augments its ability to recognize DNA damage (38,43); our *in vitro* ubiquitination assays contained UV-irradiated DNA, which significantly stimulates CRL4^DDB2^-mediated ubiquitination. Consistent results were obtained when the XPC complex was titrated in the presence of a fixed concentration of ubiquitin (Figure 7C). The competitive suppression of polyubiquitination by XPC occurred regardless of which sites in DDB2 were modified, N-terminal tail or β-propeller domain (Supplementary Figure S9). On the basis of these findings, we conclude that when XPC is recruited by UV-DDB to sites with UV-induced photolesions, it can competitively suppress poly-ubiquitination of DDB2 and thus regulate its degradation.

## DISCUSSION

Our previous studies revealed that seven lysine residues within the structurally disordered N-terminal tail of human DDB2 are the major targets of UV-induced ubiquitination by CRL4^DDB2^, and thus regulate its degradation by the proteasome (22). Here, we show that the DDB2 N-terminal tail plays an important role in cellular responses to UV. Exogenous expression of DDB2-WT conferred marked UV resistance on a normal human fibroblast cell line, but this effect was differentially abrogated by various mutations introduced into the N-terminal tail; changing all seven lysines to arginines completely abolished the UV resistance conferred by DDB2, whereas deletion of the N-terminal tail had a much smaller effect. It is notable that these exogenously expressed DDB2 proteins exerted dominant effects on cellular responses to UV even in the presence of endogenous DDB2.

Because both DDB2-Ndel and DDB2-N7KR were resistant to UV-induced degradation, the observed difference suggests additional roles for the N-terminal tail besides regulation of ubiquitination and degradation. The serine/threonine kinase p38 MAPK phosphorylates DDB2, and this modification is involved in regulation of chromatin remodeling and DDB2 stability (44). Although the exact sites targeted by p38 MAPK have not been identified, proteomic approaches revealed that at least serines 24 and 26 of DDB2 undergo phosphorylation *in vivo* (45,46). Moreover, poly(ADP-ribosyl)ation occurs at lysine residues within the N-terminal tail; this modification suppresses degradation of DDB2 and recruits the chromatin remodeling factor ALC1 (47). Taken together, these observations suggest that multiple post-translational modifications, including ubiquitination, phosphorylation and poly(ADP-ribosyl)ation, functionally interact with each other and coordinately regulate the stability of DDB2 and GG-NER overall. On the other hand, DDB2 and the tumor suppressor p53 mutually influence each other's functions (32,48,49), although the role of DDB2 in the p53-dependent apoptosis pathway remains controversial (50–55). The precise molecular mechanism underlying DDB2–p53 interactions remains to be elucidated, but it is possible that the N-terminal tail of DDB2 interacts with protein factors that mediate DNA damage signaling upstream p53. If so, the 7KR mutations in the N-terminal tail may block lysine-targeted modifications, but still allow interaction with certain proteins.

Based on the *in vitro* ubiquitination system containing CRL4^DDB2^ ligase, we previously showed that poly-ubiquitination, but not mono-ubiquitination, of DDB2 abolishes its highly specific and potent interaction with UV-damaged DNA (30). Although repair of UV-induced photolesions is stimulated by UV-DDB *in vivo*, the impact of UV-DDB on the cell-free NER reaction remains somewhat obscure; in particular, in the absence of ubiquitination, UV-DDB inhibits *in vitro* repair of 6-4PPs, probably due to its extraordinarily strong binding to this type of lesion (30,56,57). Because the affinities of UV-DDB and XPC for DNA photolesions are quite different, we hypothesized that poly-ubiquitination of DDB2 may stimulate its dissociation from a photolesion and, consequently, promote handover of damage to XPC (30,36). In addition to the ubiquitination sites in the N-terminal tail, we identified additional potential sites in the β-propeller domain of DDB2, and demonstrated that poly-ubiquitination abrogated the damaged DNA-binding activity of DDB2 regardless of which sites were modified. Based on these results, we generated a novel DDB2 mutant (DDB2-Ndel/BP5KR) that was almost completely resistant to ubiquitination at least *in vitro*. When expressed in a normal human fibroblast cell line, however, this mutant protein remained bound to chromatin following UV irradiation for a longer period than other mutants, but eventually reverted to the soluble fraction. Although this observation suggests that poly-ubiquitination may not play an essential role in dissociation of DDB2 from the sites with photolesions, we cannot rule out the possibility that DDB2-Ndel/BP5KR still undergoes ubiquitination *in vivo* at cryptic sites, albeit with lower efficiency. Alternatively, unknown mechanisms may exist by which XPC can displace UV-DDB from the damaged sites, with or without the aid of other factors. Nevertheless, expression of DDB2-Ndel/BP5KR affected cellular NER functions, especially repair of 6-4PPs (Figure 4C), so that the observed persistence of the mutant DDB2 on chromatin (Figure 4E) may be associated with a defect in some early steps of NER following DNA binding by UV-DDB. Furthermore, among the isolated cell lines, DDB2-Ndel/BP5KR was expressed only at a significantly lower level than other DDB2 proteins, while expression of endogenous DDB2 seemed to be elevated instead (Figure 4A). Given that such non-ubiquitinatable DDB2 could be harmful to proliferating cells due to its rather stable association with chromatin, selection may have been made so as to compensate for the problem by up-regulating endogenous DDB2.

The most striking finding of this study was the observation that the stability of endogenously expressed DDB2 protein is positively regulated by XPC; therefore, when XPC is expressed at higher levels, UV-induced degradation of DDB2 is accordingly less pronounced. Taken together with suppression of DDB2 auto-ubiquitination *in vitro*, this finding suggests that XPC recruited to the UV-DDB-bound sites preferentially absorbs ubiquitination by the CRL4^DDB2^ ligase, and thereby protects DDB2 from ubiquitination and degradation. When XPC is not recruited in an appropriate manner, CRL4^DDB2^ may be forced to target DDB2, which is then targeted for degradation. This model is of substantial biological relevance because it answers a longstanding question regarding why DDB2 needs to be degraded after a damage-recognition event despite the persistence of unrepaired photolesions. Many studies, including those of our group, have reported UV-induced degradation of ectopically expressed, tagged DDB2. Because the model delineated above implies that the molar balance between DDB2 and XPC is a critical factor affecting stability of DDB2, one can assume that overexpression of DDB2 itself evokes the overall destabilization of the protein. Moreover, as shown in Figure 6D, the intensity of endogenous (unmodified) DDB2 bands decreases transiently after UV irradiation even in the presence of overexpressed XPC. This observation suggests that protection by XPC of DDB2 from ubiquitination may not be complete, whereas the level of such DDB2 ubiquitination is nonetheless insufficient for recognition by the proteasome; thus deubiquitination may play a role in regulation of DDB2 as well as XPC, as reported recently (37). Despite *in vivo* evidence for recruitment of XPC by UV-DDB, assembly of the two damage-recognition factors on a single DNA lesion has not been demonstrated *in vitro*. Further biochemical and structural studies are necessary to elucidate how XPC suppresses ubiquitination of DDB2.

According to our model, it is preferable for activation of the CRL4^DDB2^ ligase to be suppressed until XPC is recruited. Previously, activation of the Cullin-based E3 ligase families was suggested to involve multiple steps of regulation (58). Our previous study indicated that the COP9 signalosome, an eight-subunit regulatory complex that suppresses ligase activity (59,60), is sterically displaced by the interaction of CRL4^DDB2^ with damaged DNA (22). Another critical factor in this process is conjugation of Cullins to the ubiquitin-like protein NEDD8. This modification induces a structural change in the Cullin–RBX1 subcomplex, causing it to adopt an opened conformation, by which the catalytic center for ubiquitination acquires a more flexible configuration; this structural alteration, in turn, prevents interaction with CAND1, another inhibitor of Cullins (61). Although the mechanism underlying regulation of CUL4 NEDDylation in CRL4^DDB2^ remains to be understood, the presence or absence of XPC may affect this process, and thereby modulate the spatial range and substrate selectivity of ubiquitination. On the other hand, in the absence of XPC, full activation of CRL4^DDB2^ may not occur promptly upon DNA binding, whereas the N-terminal tail of DDB2 is eventually ubiquitinated, leading to release of DDB2 from the damage site and its subsequent degradation. Our cell-free system may not be appropriate for investigations of these regulatory mechanisms, because the purified recombinant CRL4^DDB2^ ligase is constitutively active, and XPC probably interacts only with a portion of the DDB2 included in the reaction. Further studies using a more refined system that recapitulates *in vivo* regulation should shed light on these issues.

## SUPPLEMENTARY DATA

Supplementary Data are available at NAR Online.

SUPPLEMENTARY DATA
