# Supplementary material for: Functional regulation of the DNA damage-recognition factor DDB2 by ubiquitination and interaction with xeroderma pigmentosum group C protein
Source: Nucleic Acids Res. 2015 Jan 27;43(3):1700–13. doi: 10.1093/nar/gkv038 (PMC4330392; doi:10.1093/nar/gkv038)
Supplement: SUPPLEMENTARY DATA [file supp_43_3_1700__index.html]

Functional regulation of the DNA damage-recognition factor DDB2 by ubiquitination and interaction with xeroderma pigmentosum group C protein — SUPPLEMENTARY DATA 

# Functional regulation of the DNA damage-recognition factor DDB2 by ubiquitination and interaction with xeroderma pigmentosum group C protein

## SUPPLEMENTARY DATA

**Files in this Data Supplement:**

- Supplementary Figures
