# Supplementary material for: Functional regulation of the DNA damage-recognition factor DDB2 by ubiquitination and interaction with xeroderma pigmentosum group C protein
Source: Nucleic Acids Res. 2015 Jan 27;43(3):1700–13. doi: 10.1093/nar/gkv038 (PMC4330392; doi:10.1093/nar/gkv038)
Supplement: SUPPLEMENTARY DATA [file supp_gkv038_nar-02877-d-2014-File009.pdf]

SUPPLEMENTARY INFORMATION

Functional regulation of DNA damage recognition factor DDB2 through ubiquitination and interaction with xeroderma pigmentosum group C protein

Syota Matsumoto, Eric S. Fischer, Takeshi Yasuda, Naoshi Dohmae, Shigenori Iwai, Toshio Mori, Ryotaro Nishi, Ken-ichi Yoshino, Wataru Sakai, Fumio Hanaoka, Nicolas H. Thomä & Kaoru Sugasawa

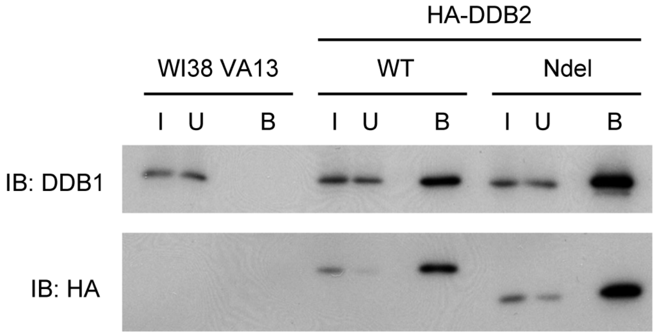

Supplementary Figure S1. The N-terminal tail of DDB2 is not required for interaction with DDB1. Exogenously expressed HA-DDB2 (WT or Ndel) was immunoprecipitated with the anti-HA antibody from the corresponding cell extract, and the bound proteins (B) were subjected to immunoblotting, together with 3% each of the input extracts (I) and the unbound fractions (U). The parental WI38 VA13 cells were used as a control.

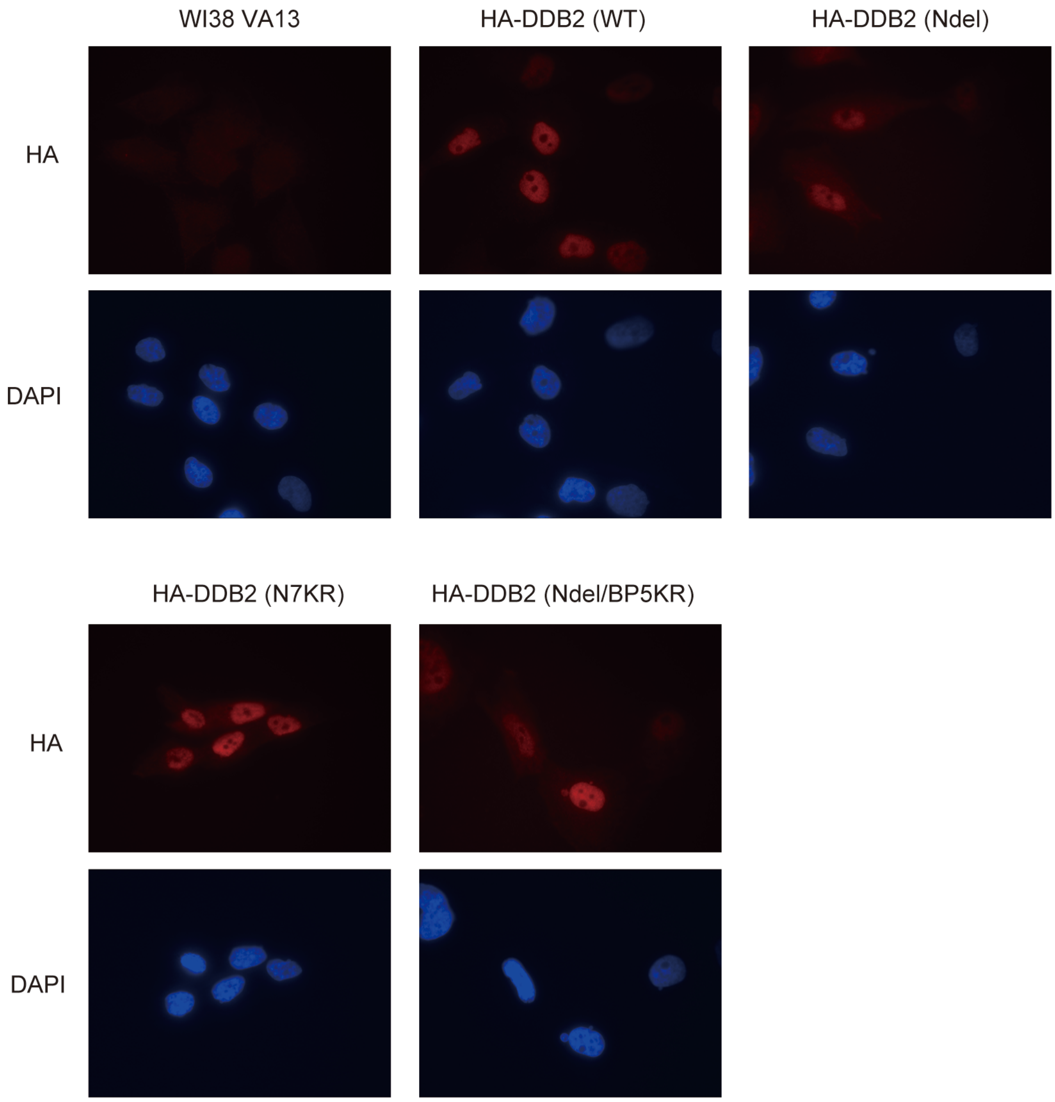

Supplementary Figure S2. Subcellular localization of exogenously expressed HA-DDB2 proteins. WI38 VA13 cells and the transformed cell lines stably expressing various HA-DDB2 proteins were subjected to immunofluorescence staining with the anti-HA antibody (red). 4',6-Diamidino-2-phenylindole (DAPI) was used to counterstain the nuclei (blue).

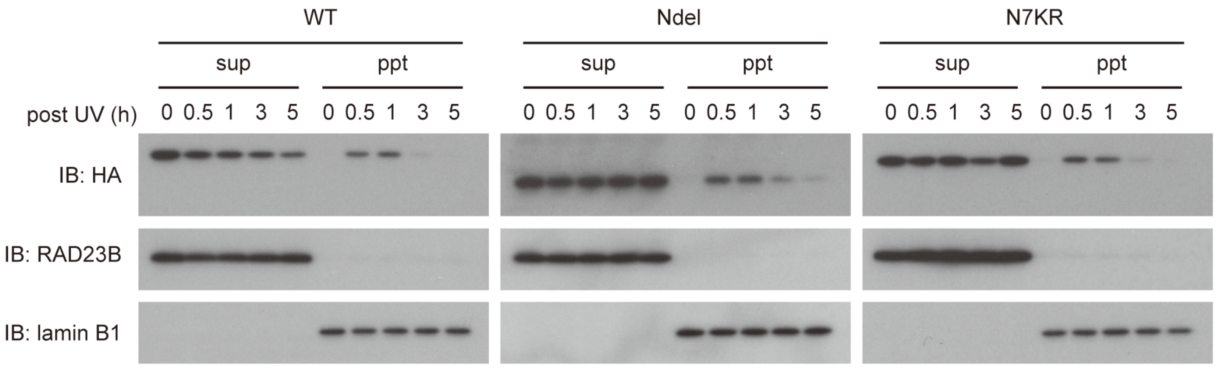

Supplementary Figure S3. Behaviors of mutant DDB2 proteins lacking the N-terminal lysines in response to UV irradiation. The transformed cell lines expressing indicated HA-DDB2 (WT, Ndel, or N7KR) were irradiated with UVC at 10 J/m^2. After further incubation for various times, the cells were fractionated into soluble extracts (sup) and insoluble materials (ppt), which were then subjected to immunoblot analyses. This is a similar experiment as Figure 2, except that the treatment with cycloheximide was omitted.

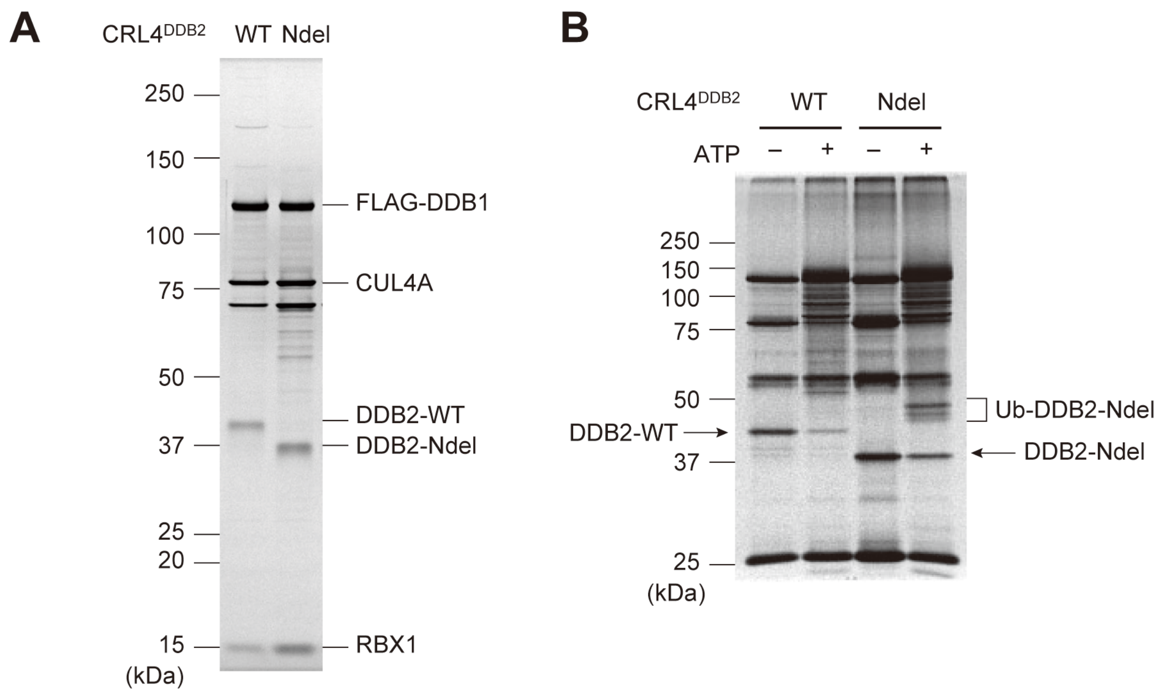

Supplementary Figure S4. *In vitro* ubiquitination reactions with the CRL4 E3 complex containing DDB2-Ndel. (A) Purified CRL4 E3 complex containing either DDB2-WT or DDB2-Ndel was subjected to SDS-PAGE followed by silver staining. (B) The CRL4^{DDB2} complexes shown in (A) were immobilized on anti-FLAG beads, and used for *in vitro* ubiquitination reactions with K-less ubiquitin in the presence (+) or absence (-) of ATP. After an extensive wash, bound proteins were eluted with FLAG peptide, and subjected to SDS-PAGE followed by silver staining.

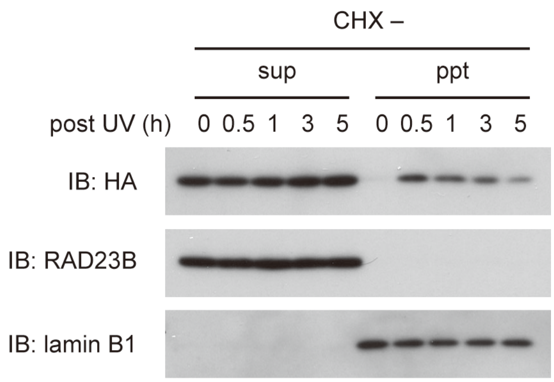

Supplementary Figure S5. Behaviors of the DDB2-Ndel/BP5KR protein in response to UV irradiation. The transformed cell line from WI38 VA13, stably expressing HA-DDB2-Ndel/BP5KR, was treated and analyzed as in Figure 4D, except that cycloheximide was omitted from the culture.

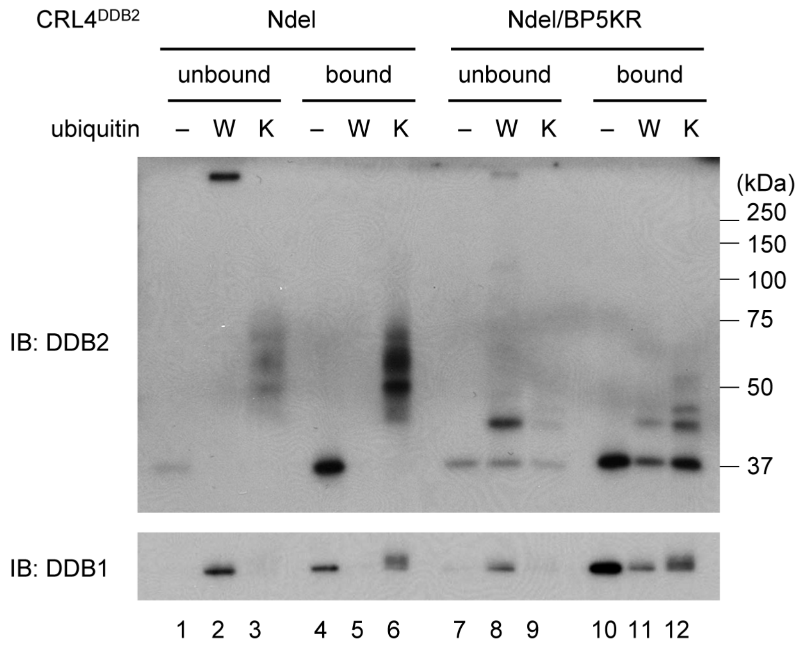

Supplementary Figure S6. Damaged DNA binding assays with *in vitro* ubiquitinated DDB2-Ndel and -Ndel/BP5KR. The CRL4 E3 ligase complex containing the indicated DDB2 was used for *in vitro* ubiquitination in the presence of paramagnetic beads bearing DNA containing 6-4PPs as done in Figure 5. Reactions were performed in the absence of ubiquitin (-), or in the presence of wild-type ubiquitin (W) or K-less ubiquitin (K). Proteins bound or unbound to the DNA beads were separated and subjected to immunoblot analyses.

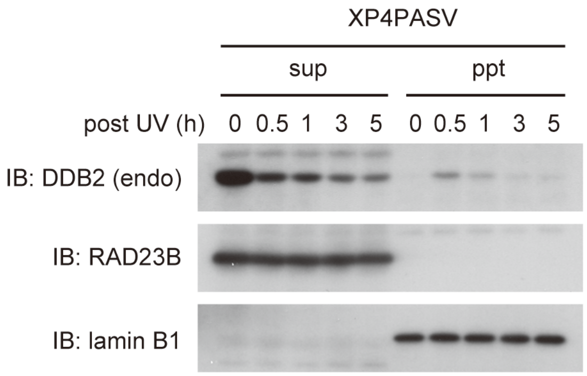

Supplementary Figure S7. UV-induced instability of the endogenous DDB2 protein in the absence of XPC. The *XPC*-deficient, XP4PASV cells were treated and analyzed as in Figure 6A, except that cycloheximide was omitted from the culture.

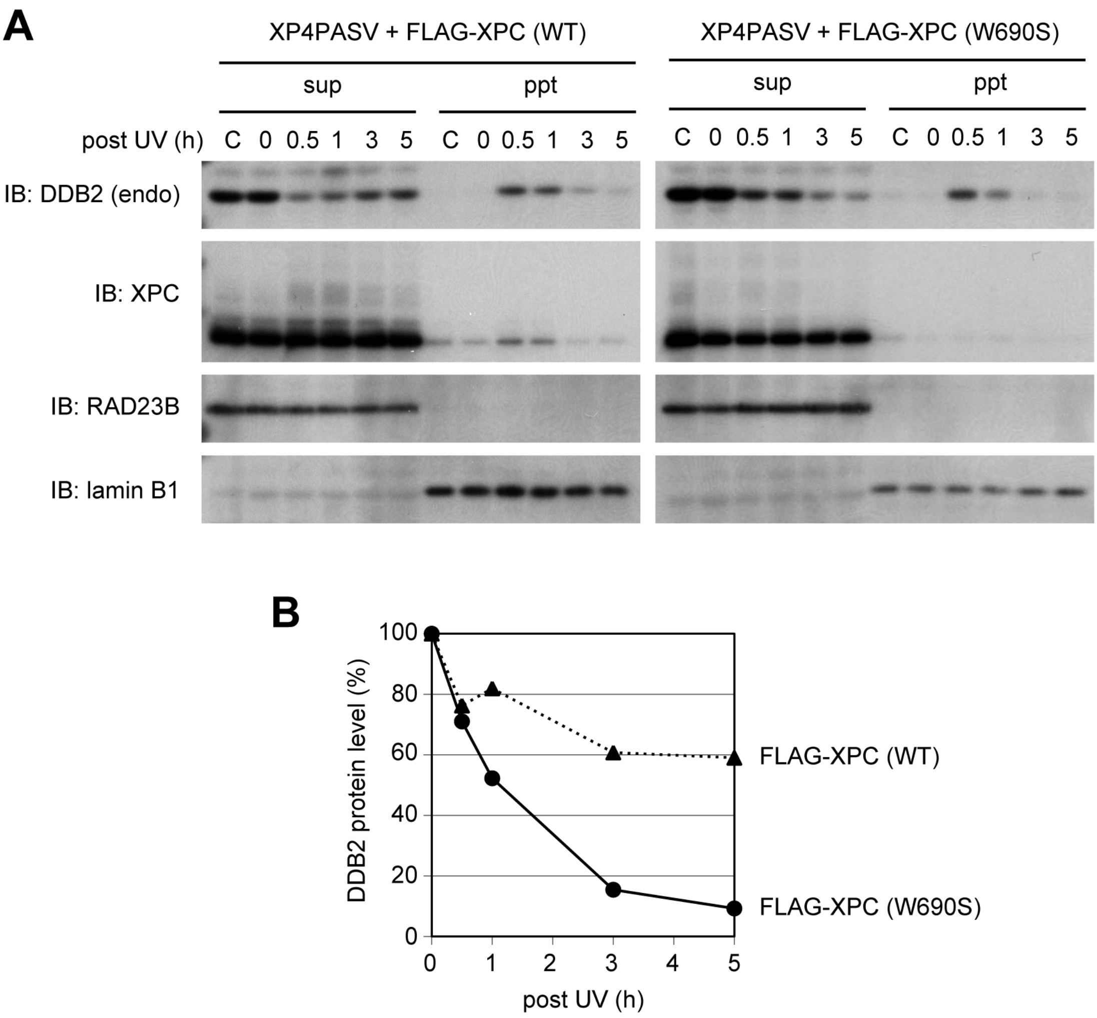

Supplementary Figure S8. The DNA binding activity of XPC is crucial for protection of DDB2 from UV-induced destabilization. (A) The XP4PASV-derived cell lines stably expressing FLAG-XPC, WT or W690S mutant, at nearly physiological levels were treated with cycloheximide (CHX), irradiated with UV-C, and fractionated as done in Figure 2. As a control, cells without CHX and UV treatment were analyzed in parallel (indicated by 'C' above the lanes). (B) From the results in (A), relative amounts of the endogenous DDB2 protein were quantified and plotted as done in Figure 6D.

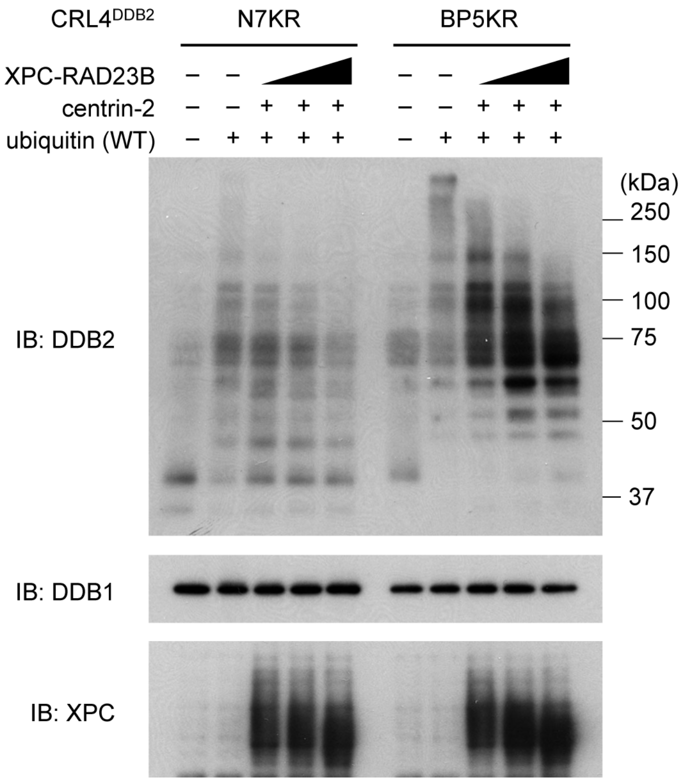

Supplementary Figure S9. Poly-ubiquitination of DDB2 is suppressed by XPC regardless of the modification sites. *In vitro* ubiquitination reactions were performed with the CRL4^{DDB2} complex (63 ng) containing either DDB2-N7KR or -BP5KR. Ubiquitin (125 ng), centrin-2 (330 ng) and varied amounts of XPC-RAD23B (82, 164, 328 ng) were included where indicated. After incubation, the reaction mixtures were subjected to immunoblot analyses with the indicated antibodies.
